# Recent advances in understanding dengue

**DOI:** 10.12688/f1000research.6233.1

**Published:** 2016-01-19

**Authors:** Sophie Yacoub, Juthathip Mongkolsapaya, Gavin Screaton

**Affiliations:** 1 Department of medicine, Imperial College London, London, UK; 2 Oxford University Clinical research Unit, Wellcome Trust Major Overseas Programme, Ho Chi Minh City, Vietnam; 3 Dengue Hemorrhagic Fever Research Unit, Office for Research and Development, Siriraj Hospital, Mahidol University, Bangkok, Thailand

**Keywords:** dengue, dengue virus, Flavivirus

## Abstract

Dengue is an emerging threat to billions of people worldwide. In the last 20 years, the incidence has increased four-fold and this trend appears to be continuing. Caused by one of four viral serotypes, dengue can present as a wide range of clinical phenotypes with the severe end of the spectrum being defined by a syndrome of capillary leak, coagulopathy, and organ impairment. The pathogenesis of severe disease is thought to be in part immune mediated, but the exact mechanisms remain to be defined. The current treatment of dengue relies on supportive measures with no licensed therapeutics available to date. There have been recent advances in our understanding of a number of areas of dengue research, of which the following will be discussed in this review: the drivers behind the global dengue pandemic, viral structure and epitope binding, risk factors for severe disease and its pathogenesis, as well as the findings of recent clinical trials including therapeutics and vaccines. We conclude with current and future dengue control measures and key areas for future research.

## Introduction

Dengue has emerged in the last two decades as the most abundant vector-borne viral infection globally. The dengue virus belongs to the Flavivirus family and has four serotypes (DENV1-4), which are clinically indistinguishable. Latest estimates suggest 390 million infections of dengue occur each year, of which 100 million result in symptomatic disease ^1^. Dengue may present as a spectrum of clinical syndromes from dengue fever, a non-specific febrile illness, through to severe dengue ( Box 1) (replacing the original classification of dengue fever/dengue hemorrhagic fever [DHF] ^2^). The 2009 World Health Organization (WHO) dengue guidelines have outlined a number of warning signs ( Box 2) to assist triaging the often vast numbers of patients that can present to clinics in endemic areas; however, the ability to predict which patients will progress to severe disease remains challenging.

Box 1. **Criteria for severe dengue**

• Severe plasma leakage leading to
  1) Shock and/or
  2) Fluid accumulation with respiratory distress
• Severe bleeding
• Severe organ involvement
  Liver: alanine transaminase or aspartate aminotransferase >=1000
  Central nervous system: impaired consciousness
  Heart and other organs

Box 2. **Criteria for dengue warning signs, taken from the 2009 WHO classification**

- Abdominal pain or tenderness
- Persistent vomiting
- Clinical fluid accumulation
- Mucosal bleed
- Lethargy/restlessness
- Liver enlargement >2 cm
- Laboratory increase in hematocrit concurrent with rapid decrease in platelet count

One of the defining features of severe disease is increased capillary permeability causing plasma leakage, which can lead to intravascular volume depletion and, if left untreated, shock and death. The underlying mechanisms for progressing to severe disease have not been fully elucidated, but due to the strong association of severe dengue and secondary infection with a different serotype, an immune-mediated pathogenesis has been postulated. Both T-cell-mediated immunopathogenesis ^3^ and antibody-dependent enhancement (ADE) have been implicated ^4^. Because of the potential for more severe outcome in sequential infections, developing a safe and balanced vaccine for all four serotypes has been challenging ^5^.

This review will focus on recent advances in understanding the drivers of the dengue pandemic, viral structure and epitope binding, clinical severity and potential risk factors, plus recent studies investigating the pathogenesis and therapeutic options, concluding with strategies for disease control and future directions.

## Global expansion and disease burden

Over the past 30 years, there has been a huge expansion in the transmission of dengue, and currently it is endemic in more than 100 countries ^2^. Over 70% of the global burden lies in South and South-East Asia, but more recently case numbers have exploded in other parts of Asia, Latin America, and the Caribbean. Although harder to quantify, the African continent has also witnessed a significant increase in cases, with outbreaks reported from a number of East and West African countries ^6, 7^. It has also become apparent in recent years that developed countries are at risk, with small outbreaks being reported more from Southern Europe, the USA, and northern Australia. In 2012, Europe experienced its first dengue epidemic since the 1920s when over 2000 cases and 120 hospital admissions were reported from the Portuguese island of Madeira ^8^. The origin of this outbreak was most likely from a viremic traveler from Venezuela, taking into account the volume of travel to Madeira from dengue endemic countries, seasonality in these countries, and also genetically similar viruses circulating in Venezuela at the time of the outbreak ^9^. A similar although smaller outbreak occurred in Japan in 2014, again thought to have involved a viremic traveler with ongoing autochthonous spread associated with a large park in Tokyo ^10^.

Overall, the drivers behind the global expansion in disease are thought to include certain vector and host factors, including the urban-adapted *Aedes* mosquito vector becoming newly established in many areas of the world through distribution on cargo ships, globalization, and increase in breeding sites through rapid and often poorly planned urbanization of cities ^11^. Other suggested factors include climate change and increase in population mobility and air travel ^12, 13^. These factors combined with ineffective vector control programs and no licensed therapeutics or vaccines has meant dengue is now a public health threat for two-thirds of the world’s population.

## Viral structure and epitope binding

The dengue virus is a single-stranded, positive-sense enveloped RNA virus, 50 nm in diameter. The dengue virus genome encodes three structural proteins (capsid [C], precursor membrane [prM], and envelope [E]) and seven non-structural proteins (NS1, NS2A, NS2B, NS3, NS4A, NS4B, and NS5).

Studies using cell culture have shown prM and E insert into the virion membrane to form the glycoprotein shell of the virus. During viral production and assembly, there is a complex series of rearrangements of prM and E. The virus is assembled in the endoplasmic reticulum, where 180 copies of both prM and E associate into trimeric spikes, each containing three prM and three E proteins ^14^. prM acts as a chaperone protecting the hydrophobic fusion loop of E from triggering premature fusion with host cell membranes. As the virion traffics through the Golgi, furin protease cleaves prM, and as the virion is secreted from the cell the cleaved pr polypeptide is released and the E protein rearranges into 90 dimers, giving a smooth mature virus particle ^15^. Following adhesion to poorly characterized cellular receptors, the virus is endocytosed and acidification of the endocytic vesicle then triggers E to reassociate from dimers to trimers, which exposes the fusion loop, allowing the virion to fuse with the endocytic membrane, releasing the viral RNA into the host cell cytoplasm ^16^. One further complication of this is that furin cleavage of prM is often incomplete, leading to the production of virions with varying amounts of cleaved and uncleaved prM ^17, 18^.

The E protein has three domains (DI-III), is required for receptor binding and cell fusion and entry ^19^, and is the major target for neutralizing antibodies, with potent neutralizing mouse monoclonal antibodies binding to epitopes on the DIII region ^20, 21^. The most potent human antibodies appear to bind to conformationally sensitive epitopes that are only found on intact virions and not with denatured or monomeric E protein ^22^. It is now clear that the binding of some antibodies is limited by the accessibility of their epitopes, and that breathing of the virion and conformational change in the arrangement of E in the virion lattice may be required for binding ^23^. In addition, broadly neutralizing anti E monoclonal antibodies directed at DII have been found to increase their avidity following secondary infection ^24^. There are a number of serotype-specific human monoclonal antibodies which also recognize quaternary epitopes: HM14C10, 5J7, and 1F4 bind epitopes across three adjacent E monomers, whilst 2D22 binds across the E dimer ^25– 28^. Antibodies to prM are produced at high levels following dengue infection, but they are very poor at neutralizing infection, reaching a threshold of activity with none able to fully neutralize infection ^29^. During the process of viral maturation, prM is cleaved, so anti-prM antibodies may fail to neutralize many viral particles because the antibody binding threshold required for neutralization will not be met. As mentioned above, the cleavage of prM is, however, frequently incomplete, which means that many virions contain enough prM to drive ADE but insufficient to promote neutralization. In addition, immature viruses which are usually non-infectious and which have a high density of uncleaved prM can become infectious to cells via ADE ^17, 29^.

An exciting recent development by our group is the discovery of a new class of antibodies directed at a novel epitope: the E dimer epitope (EDE), which is capable of potently neutralizing all four dengue serotypes ^30^. The structure of these broadly neutralizing antibodies was characterized using X-ray crystallography and cryo-electron microscopy, and revealed that they recognized a serotype-invariant site that is located at the E-dimer interface, which includes contacts to the main chain of the E fusion loop ^31^. This is also the binding site of prM during viral maturation, as previously described ^32^. This has major implications for the future development of a subunit vaccine.

Other advances have recently been made in determining the structure and function of the NS1 protein. NS1 is a 50 kDa glycoprotein that is secreted from dengue-infected cells and can be detected in the patient’s serum from early in the disease through to several days after defervescence. NS1 may play a role in the pathogenesis of severe disease, as higher levels have been detected in dengue shock patients ^33^. Further work has identified that NS1 is secreted from infected cells as a hexamer, which creates a barrel shape around a lipid core ^34^, and using cryo-electron microscopy the assembly and antibody binding of NS1 have also been described ^35, 36^. Antibodies against NS1 may be a potential therapeutic target, and modified NS1 may provide an alternative vaccine strategy ^37^.

## Clinical severity and risk prediction

The severe manifestations that develop in a small proportion of dengue-infected patients occur relatively late in the course of the illness, usually day 4–6, at the time of fever clearance. The most common severe manifestation is vascular leakage, which can lead to hemodynamic compromise, shock, and death. In addition, bleeding from mucosal surfaces and organ impairment in the form of hepatitis, myocarditis, and encephalitis can occur. This 48-hour period around defervescence has been classed as the “critical phase” and is the time when patients require closer monitoring. The WHO updated their classification and guidelines in 2009 to incorporate a set of warning signs to identify higher risk patients. These include a set of signs and symptoms and laboratory parameters to guide clinicians as to which patients are at a higher risk for disease progression ( Box 2). In addition, several studies have identified certain risk factors that can influence disease severity in dengue; these include specific host and viral factors that likely act in concert to determine the disease phenotype ^38^.

Viral factors include both the infecting serotype and the genotype of the virus, with certain genotypes within each serotype considered more virulent than the others, and have been linked to outbreaks of severe disease ^39, 40^. Higher viral loads have been associated with disease severity in both primary and secondary dengue and with different serotypes ^41, 42^.

 The underlying immune status of the host is one of the most important factors in determining disease outcome, with a primed immune response, under certain conditions, facilitating a higher viral infected cell mass through ADE ^43^ and original antigenic sin ^44^, which will be discussed further in the pathogenesis section below. Other host factors include age of the host, with children more likely to experience plasma leakage and shock, and adults more likely to develop organ impairment and significant bleeding ^45^. Elderly patients and those with co-morbidities, including diabetes and hypertension, have also been found to be at an increased risk of severe dengue ^46^, possibly due to pre-existing endothelial dysfunction in this group. Female sex and age of less than 5 years have also been identified as risk factors for poor outcomes ^47^. Genetic predisposition is also likely to play a role, with a genome-wide association study in Vietnam identifying two loci that were associated with severe disease, MICB and PLCE1 ^48^, and a further study confirming these loci were also associated with less severe forms of dengue, as well as with dengue in infants ^49^. Other genetic factors that have been shown to affect disease severity include certain HLA alleles, variations in the vitamin D receptor and Fc gamma receptor IIa, and also CD209 (G allele variant of DCSIGN1-336) ^50– 52^.

## Pathogenesis of severe disease

There have been several recent advances in understanding dengue’s pathogenesis; however, the exact mechanisms remain to be fully defined. The observation that severe dengue occurs more frequently in secondary infections may be explained by ADE, where heterotypic non-neutralizing antibodies from a previous dengue infection facilitate viral binding to Fc receptors of monocytes and macrophages, leading to higher viral loads and more marker inflammatory response ^43^. In addition, cross-reactive memory T cells also appear to play an important role in triggering the inflammatory cascade. The exact role of CD8+ T cells in the pathogenesis of severe disease is a rapidly evolving field, with some studies suggesting a pathogenic role with higher frequencies of cross-reactive CD8+ T cells being found in severe disease during secondary infections. Cells of low affinity for the infecting virus but higher affinity for other, presumed previous serotypes may be less effective at clearing the infection, resulting in a higher viremia. However, other studies suggest an HLA-linked protective role of CD8+ cells with a robust multifunctional response being associated with less severe disease ^53^. Further work has demonstrated the T cell response was most marked to NS3 protein, with high cytokine and low CD107a (a marker of cell degranulation) predominating ^54^. The resulting cytokine release, particularly tumor necrosis factor alpha and other vasoactive mediators, may then play a role in the increase in capillary permeability seen in severe dengue ^55– 57^.

The mechanisms linking these immunopathogenesis studies to vascular injury are still lacking. NS1 has been implicated in the pathogenesis of vascular leak. High levels of the soluble NS1 have been identified in patients’ plasma, from early in the disease and for up to 2 weeks later ^41^, and like the viral load, NS1 antigenemia appears to correlate with disease severity ^33^. NS1, along with the viral E protein, are able to bind to heparan sulfate, one of the major glycosaminoglycans (GAGs) in the glycocalyx of the endothelial cell layer ^58, 59^. The glycocalyx consists of a negatively charged network of glycoproteins, proteoglycans, and GAGs that covers the luminal surface of the microvascular endothelium. It provides size and charge selectivity to the capillary wall permeability, as well as acting as a transducer of sheer stress ^60^. The adherence of NS1 and of the DENV E protein to the glycocalyx, and the resulting damage, could alter the permeability properties of the microvascular layer, which may contribute to the characteristic vascular leak that is associated with severe dengue ^58, 59, 61^.

NS1 and anti-NS1 antibodies have also been implicated in the pathogenesis of thrombocytopenia and coagulopathy that is characteristic in dengue ^62, 63^. NS1 can also activate complement, which may contribute to the vascular leak through the generation of anaphylatoxins and the terminal complement complex SC5b-9 ^59^. High plasma levels of NS1 and SC5b-9 in dengue patients correlated with disease severity, and were also detected along with the anaphylatoxin C5a in the pleural fluid of dengue shock patients. In addition, anti-NS1 antibodies have been implicated in complement-mediated cytolysis and endothelial cell damage ^64, 65^. Recent *in vitro* studies have demonstrated that NS1 can alter endothelial monolayer integrity through the activation of Toll-like receptor 4 on peripheral blood mononuclear cells ^66^, and altered endothelial permeability was prevented in mice by blocking NS1 through vaccination and monoclonal antibodies to NS1 ^67^.

Other immunological parameters that may play a role in the pathogenesis of severe dengue include plasmablast frequency, with high levels correlating with the critical phase ^68^, mast cell activation and mast-cell-derived mediators, particularly vascular endothelial growth factor ^69, 70^, and antibody-immune complexes ^71, 72^.

## Current and novel therapeutic options

The current management of dengue relies on supportive treatment in the form of close monitoring for any of the “warning signs” and careful fluid balance for those identified to have capillary leak. Intravenous fluid is usually only required for patients with significant vascular leak and hemodynamic instability, or patients unable to tolerate oral fluids. The current WHO management guidelines recommend the initial use of crystalloid solutions, followed by colloid solutions for patients with profound or unresponsive shock ^2^. Further trials are required to investigate whether earlier intervention with a colloid solution would benefit patients with dengue shock. Also fluid management in adult/elderly patients and those with co-morbidities is required, as evidence from randomized controlled trials in these groups are lacking.

There have been several disappointing therapeutic trials in dengue investigating both antivirals and adjunctive therapies. Two recent antiviral trials studying balapiravir in Vietnam and celgosivir in Singapore failed to demonstrate any beneficial effect on viremia or clinical outcome ^73, 74^.

In addition, adjunctive therapies have yet to demonstrate any disease-modifying effect. The anti-malarial drug chloroquine, although it showed promising antiviral effects *in vitro* ^75^, did not translate into a reduction in viremia or NS1 duration in a randomized controlled trial in adult dengue patients ^76^. Immunomodulation with corticosteroids has also failed to alter disease severity both in patients with established dengue shock and also when given early in the disease course ^77, 78^. A study using intravenous immunoglobulin did not impact on the development of severe thrombocytopenia ^79^, nor did prophylactic platelet transfusions have any benefit on bleeding manifestations in adult patients with severe thrombocytopenia ^80^.

*In vitro* studies have shown lovastatin is able to interrupt the DENV assembly pathway ^81^ and increase survival in animal models ^82^. A human study investigating lovastatin in early dengue has just been published, again showing no benefit in modifying dengue clinical outcomes ^83^. It is anticipated that dengue drug discovery in the next few years will be assisted by improved animal models for dengue ^84^ and also the possibility of a human infection model ^85^.

## New strategies for dengue control

Efforts to control the spread of dengue in the last two decades have failed, mainly due to the lack of a licensed vaccine and difficulties in controlling the major global vectors *Aedes aegypti* mosquitoes and, more recently, *Aedes albopictus* ^86^. These day-biting, anthropophilic mosquitoes are highly adapted to the urban environment, breeding primarily in man-made water containers. Previously, vector control efforts were aimed at the elimination of the container breeding sites, improved access to piped water supplies, and improved management of water storage. The use of larvicides and insecticides were mainly used during outbreaks and had many limitations, including resistance ^87^. However, new technologies showing some promise for future dengue control are biologic and genetic modification of mosquitoes. The intracellular bacterium *Wolbachia*, when introduced into *Aedes* mosquitoes, can influence the ability of the insects to transmit the virus, indirectly by reducing the mosquito’s life span and directly by reducing viral replication in the mosquito ^88, 89^. Field trials are underway in Vietnam ^90^ and Australia ^91^ (also Brazil and Indonesia), and have demonstrated successful invasion of *Wolbachia*-infected mosquitoes into natural mosquito populations at the release sites ^92^. In addition, there are some promising results of genetically manipulated mosquitoes with engineered sterile male *Aedes* mosquitoes in field trials in the Cayman Islands ^93^.

Although currently there is no global dengue vaccine available for public health use, in the last 2 months the first ever dengue vaccine (CYD-TDV, Sanofi-Pasteur) was licensed in Mexico followed by the Philippines, in addition there are several candidate vaccines in different phases of development, e.g. live attenuated, inactivated whole virus, and subunit and recombinant vaccines. Several live attenuated vaccines have progressed to clinical trials, but the concern for ADE with an unbalanced response to all four serotypes has been a major challenge. The lead candidate and recently licensed in 2 countries is a tetravalent live attenuated vaccine (CYD-TDV, Sanofi-Pasteur) has recently completed the first phase III dengue vaccine trial in Asia ^94^ and Latin America ^95^. The overall vaccine efficacy was 56.5% in Asian children and 64.7% in slightly older children in Latin America. However, this varied by serotype, with poor efficacy for DENV-2 of only 35% in the Asian study and 42.3% in Latin America, and also varied depending on background flavivirus immunity, with poor efficacy demonstrated in flavivirus-naive people. A further study has recently been published reporting the results of the first long-term follow up (3 years post vaccination) of the CYD-TDV vaccine and has shown continued benefit in vaccinated children aged 9–16 years. However, in the younger age group (<9 years), there was an increase in hospitalization when compared to unvaccinated subjects ^96^.

These studies have also highlighted the need to improve our understanding of the immunological correlates of disease, as neutralizing antibodies to all four serotypes were demonstrated among vaccines in an earlier phase of the study but did not translate to equal protection.

Other live attenuated vaccine candidates have reported promising results from phase 1 trials, including NIH Δ30 and DENVax from Takeda ^97– 99^. DENV-1, -3, and -4 of NIH Δ30 candidate were attenuated by deleting 30 nucleotides at the 3’ untranslated region of the viral genome, while DENV-2 was generated by replacing the DENV-4 prM and E genes with those from DENV-2. DENVax is a live attenuated DENV-2 backbone with three recombinant vaccine viruses (serotypes 1, 3, and 4) expressing prM and E genes ^98^.

Whole inactivated tetravalent vaccines may offer a safer alternative strategy, and a recent study in macaques demonstrated good immunogenicity when the vaccine was combined with an adjuvant ^100^. Subunit vaccines using the DENV E protein (domain III) as the major immunogen have shown potential in preclinical trials ^101^, and a subunit vaccine (DEN-80E) developed by Merck has now progressed to clinical trials ^102^. With the recent identification of a conserved epitope on the E protein (EDE), this is an area that is likely to develop further in the future ^30^. As with testing novel dengue therapeutics, vaccine efficacy studies should also benefit from potential human infection models in the near future ^103^.

## Conclusion and future direction

Dengue is one of the world’s most rapidly emerging diseases, and as incidence continues to rise in endemic areas, and transmission in new regions of the world becomes established, there are major public health challenges ahead. There have been recent advances in our understanding of the epidemiology, risk factors for severe disease, and pathogenesis, plus the identification of therapeutic targets, which may lead to novel treatments. Improved animal and human infection models should lead to better understanding of disease evolution and assist drug development. In addition, the advance in the study of human monoclonal antibodies has opened up a new avenue for vaccine development, which should concentrate on inducing the potent neutralizing anti-EDE antibodies against all four serotypes and avoid anti-prM antibodies, which have low neutralizing activity and high potential to enhance viral infection through ADE. Future randomized controlled trials of novel therapeutics and fluids, including in adults, will be required to guide evidence-based practice in all patient groups. With the possibility that the first ever dengue vaccine may be licensed in more countries in the next couple of years, and the further deployment of *Wolbachia* bio-control, reversing the spread of dengue may now be a real prospect.
